# Supplementary material for: Early cancer screening surveillance in one medical center of China
Source: PeerJ. 2024 Sep 27;12:e18179. doi: 10.7717/peerj.18179 (PMC11441387; doi:10.7717/peerj.18179)
Supplement: Supplemental Information 2 [file peerj-12-18179-s002.docx]

| ID | 病历号 | ID |  |  |  |  |  |  |  |  |  |  |  |
| --- | --- | --- | --- | --- | --- | --- | --- | --- | --- | --- | --- | --- | --- |
| Gender | 性别 | Gender | 1 男 Male | 2 女 Female |  |  |  |  |  |  |  |  |  |
| Age | 年龄 | Age |  |  |  |  |  |  |  |  |  |  |  |
| Marriage | 婚姻状况 | *Marriage* | *1 未婚 unmarried* | *2 已婚 married* | *3 离婚divorced* | *4 丧偶 widow* |  | 1 有伴侣 with partner | 2 无伴侣 without partner |  |  |  |  |
| Mfamily | 恶性肿瘤家族史 | Malignant family history | 1 有 yes | 2 无 no |  |  |  |  |  |  |  |  |  |
| Cancer | 癌症病史 | Cancer history | 1 有 yes | 2 无 no |  |  |  |  |  |  |  |  |  |
| History | 慢病史 | Other disease history, such as hypertension | 1 有（高血压，糖尿病，肝炎，结核，冠心病，脑梗）yes (Hypertension, Diabetes mellitus, Hepatitis, Tuberculosis, Coronary heart disease, Cerebral infarction) | 2 无 no |  |  |  |  |  |  |  |  |  |
| Pathology1 | 既往手术中是否送检病理 | Previous pathology diagnosis | 1 恶性 malignant | 2 良性 benign | 3 无 without |  |  |  |  |  |  |  |  |
| shsmoking | 二手烟接触史 | Second smoking history | 1 有 yes | 2 无 no |  |  |  |  |  |  |  |  |  |
| Smoking | 抽烟史 | Smoking history | 1 有 yes | 2 无 no |  |  |  |  |  |  |  |  |  |
| Drinking | 饮酒史 | Drinking history | 1 有 yes | 2 无 no |  |  |  |  |  |  |  |  |  |
| Gendo | 胃镜 | Gastric endoscopy | 1 可疑癌（均伴慢性萎缩性胃炎）Suspected malignant (all with chronic atrophic gastritis) | 2 高级别上皮内瘤变 high-grade intraepithelial neoplasia | 3 萎缩性胃炎/Barret食管 Atrophic gastritis /Barret esophagus | 4息肉 polypus | 5 溃疡 ulcer | 6 非萎缩性胃炎（包括浅表性胃炎，糜烂性胃炎）Non-atrophic gastritis (superficial gastritis, erosive gastritis) | 7 未见异常  Without abnormality | |  |  |  |
| Gendo1 | 胃镜 | Gastric endoscopy | 1 可疑恶性 Suspected malignant | 2 萎缩性胃炎/Barret食管 Atrophic gastritis /Barret esophagus | 3 其它良性病变 other benign lesions | 4 无异常 Without abnormality |  |  |  |  |  |  |  |
| Geendo1 | 胃镜 | Gastric endoscopy | 1 可疑癌 Suspected malignant | 2 非癌 Not malignant |  |  |  |  |  |  |  |  |  |
| PathologyG | 胃镜病理检查 | Gastric endoscopy pathology diagnosis | 1 腺癌 adenocarcinoma | 2 炎性/息肉 Inflammatory/polyposis | 3 鳞癌 squamous carcinoma | 4 溃疡 ulcer |  |  |  |  |  |  |  |
| ptg1 | 胃镜病理检查 | Gastric endoscopy pathology diagnosis | 1 腺癌 adenocarcinoma | 2 鳞癌 squamous carcinoma | 3 良性（炎性，溃疡）benign (Inflammatory, ulcer) | | |  |  |  |  |  |  |
| Eendo | 肠镜 | Colorectal endoscopy | 1 可疑癌 Suspected malignant | 2 高级别上皮内瘤变/LST（侧向发育性肿瘤）High-grade intraepithelial neoplasia /LST (laterally spreading tumor) | 3 腺瘤 adenocarcinoma | 4 溃疡 ulcer | 5 结节/息肉/隆起 A nodule/polyp/bulge | 6 无异常（无肿物或狭窄）No abnormalities (no mass or stenosis) | | |  |  |  |
| gendo2 | 肠镜 | Colorectal endoscopy | 1 可疑恶性 Suspected malignant | 2 腺瘤样息肉 adenomatoid polyp | 3 非腺瘤样息肉 Nonadenomatous polyps | 4 其它良性 other benign lesions | |  |  |  |  |  |  |
| geendo2 | 肠镜 | Colorectal endoscopy | 1 可疑癌 Suspected malignant | 2 非癌 Not malignant |  |  |  |  |  |  |  |  |  |
| PathologyE | 肠镜病理检查 | Colorectal endoscopy pathology diagnosis | 1 腺癌 adenocarcinoma | 2 腺瘤 adenoma | 3 炎性 Inflammatory | 4 增生性息肉 hyperplastic polyp | 5 溃疡性肠炎 ulcerative enteritis | |  |  |  |  |  |
| ptg2 | 肠镜病理检查 | Colorectal endoscopy pathology diagnosis | 1 腺癌 adenocarcinoma | 2 腺瘤 adenoma | 3 其它 Others |  |  |  |  |  |  |  |  |
| HCVIGG1 | 丙肝病毒抗体（HCV-IgG） | HCV antibody |  |  |  |  |  |  |  |  |  |  |  |
| HCVIGG2 |  |  | 1 阳性 positive | 2 阴性 negative |  |  |  |  |  |  |  |  |  |
| HBSAG1 | 乙肝表面抗原（HBsAg） | HBsAg |  |  |  |  |  |  |  |  |  |  |  |
| HBSAG2 |  | HBsAg | 1 阳性 positive | 2 阴性 negative |  |  |  |  |  |  |  |  |  |
| HBSAB1 | 乙肝表面抗体（HBsAb） | HBsAb |  |  |  |  |  |  |  |  |  |  |  |
| HBSAB2 |  | HBsAb | 1 阳性 positive | 2 阴性 negative |  |  |  |  |  |  |  |  |  |
| HBEAG1 | 乙肝e抗原（HBeAg） | HBeAg |  |  |  |  |  |  |  |  |  |  |  |
| HBEAG2 |  | HBeAg | 1 阳性 positive | 2 阴性 negative |  |  |  |  |  |  |  |  |  |
| HBEAB1 | 乙肝e抗体（HBeAb） | HBeAb |  |  |  |  |  |  |  |  |  |  |  |
| HBEAB2 |  | HBeAb | 1 阳性 positive | 2 阴性 negative |  |  |  |  |  |  |  |  |  |
| HBCAB1 | 乙肝核心抗体（HBcAb） | HBcAb |  |  |  |  |  |  |  |  |  |  |  |
| HBCAB2 |  | HBcAb | 1 阳性 positive | 2 阴性 negative |  |  |  |  |  |  |  |  |  |
| AntiTP | 梅毒螺旋体抗体(Anti-TP) | treponema pallidum antibody | 1 阳性 positive | 2 阴性 negative |  |  |  |  |  |  |  |  |  |
| CEA1 | 癌胚抗原 | CEA |  |  |  |  |  |  |  |  |  |  |  |
| CEA2 |  | CEA | 1 升高 higher than upper normal limit | 2 正常 Normal |  |  |  |  |  |  |  |  |  |
| CA125_1 | 肿瘤相关抗原125 | CA125 |  |  |  |  |  |  |  |  |  |  |  |
| CA125_2 |  | CA125 |  |  |  |  |  |  |  |  |  |  |  |
| CA153_1 | 肿瘤相关抗原15-3 | CA153 |  |  |  |  |  |  |  |  |  |  |  |
| CA153_2 |  | CA153 |  |  |  |  |  |  |  |  |  |  |  |
| CA199_1 | 肿瘤相关抗原19-9 | CA199 |  |  |  |  |  |  |  |  |  |  |  |
| CA199_2 |  | CA199 |  |  |  |  |  |  |  |  |  |  |  |
| SCC1 | 磷癌相关抗原 | SCC |  |  |  |  |  |  |  |  |  |  |  |
| SCC2 |  | SCC |  |  |  |  |  |  |  |  |  |  |  |
| CA724_1 | 肿瘤相关抗原72-4 | CA724 |  |  |  |  |  |  |  |  |  |  |  |
| CA724_2 |  | CA724 |  |  |  |  |  |  |  |  |  |  |  |
| NSE1 | 神经元特异性烯醇化酶 | NSE |  |  |  |  |  |  |  |  |  |  |  |
| NSE2 |  | NSE |  |  |  |  |  |  |  |  |  |  |  |
| CYFRA211_1 | 血清骨胶素CYFRA21-1 | CYFRA211 |  |  |  |  |  |  |  |  |  |  |  |
| CYFRA211_2 |  | CYFRA211 |  |  |  |  |  |  |  |  |  |  |  |
| fPSA1 | 游离前列腺特异抗原 | fPSA |  |  |  |  |  |  |  |  |  |  |  |
| fPSA2 |  | fPSA |  |  |  |  |  |  |  |  |  |  |  |
| PSA1 | 前列腺特异性抗原检查 | tPSA |  |  |  |  |  |  |  |  |  |  |  |
| PSA2 |  | tPSA |  |  |  |  |  |  |  |  |  |  |  |
|  | 超声coding最严重的疾患 |  |  |  |  |  |  |  |  |  |  |  |  |
| Umammary | 乳腺彩超 | Breast Doppler ultrasound | 1 BI-RADS 4 | 2 BI-RADS 1-3 | 3 BI-RADS 5 | 7 双乳腺未见明显异常 without abnormalities | | |  |  |  |  |  |
| U1 | 乳腺超声 | Breast Doppler ultrasound | 1 BI-RADS 5 | 2 BI-RADS 4 | 3 其它 others |  |  |  |  |  |  |  |  |
| UB1 | 乳腺超声 | Breast Doppler ultrasound | 1 BIRADS 4 and higher | 2 低级别病变 Low-grade lesions |  |  |  |  |  |  |  |  |  |
| Uprostate | 前列腺彩超 | Prostate Doppler ultrasound | 1 前列腺无异常 without abnormalities | 2 前列腺钙化/增大 Calcification/hypertrophy | 3 前列腺增大/钙化 Calcification/hypertrophy | 4 前列腺结节 prostatic tubercle | |  |  |  |  |  |  |
| U2 | 前列腺超声 | Prostate Doppler ultrasound | 1 增大/钙化/结节 prostatic Calcification/hypertrophy/ tubercle | 2 无异常 without abnormalities |  |  |  |  |  |  |  |  |  |
| Upelvis | 子宫附件彩超 | Uterus and adnexa Doppler ultrasound | 1 子宫双附件无异常 without abnormalities | 2 子宫肌瘤/腺肌症 Uterine fibroids/adenomyosis | 3 卵巢囊肿 ovarian cyst | 4 肌瘤伴囊肿 Uterine fibroids with cysts | 5 转移瘤可能 suspected metastatic tumor | 6 畸胎瘤 teratoma | 7 卵巢囊实性占位 Ovarian cystic solid mass | |  |  |  |
| U3 | 子宫附件彩超 | Uterus and adnexa Doppler ultrasound | 1 子宫肌瘤/腺肌症 Uterine fibroids/adenomyosis | 2 卵巢囊肿 ovarian cyst | 3 肌瘤伴囊肿 Uterine fibroids with cysts | 4 畸胎瘤 teratoma | 5 囊实性占位（癌性可能）Ovarian cystic solid mass (suspected malignant) | 6 无异常 without malignancies |  |  |  |  |  |
| Uthyroid | 甲状腺彩超 | Thyroid Doppler ultrasound | 1 可疑癌/性质待定，建议FNA/TI-RADS 4及以上 TI-RADS 4 or higher | 2 未见恶性征象 without malignant | 3 甲亢待排 suspected hyperthyroidism | |  |  |  |  |  |  |  |
| U4 | 甲状腺彩超 | Thyroid Doppler ultrasound | 1 可疑癌（TI-RADS 4及以上）TI-RADS 4 or higher | 2 未见异常without abnormalities | |  |  |  |  |  |  |  |  |
| Ubelly | 肝胆胰脾彩超 | Liver, gallbladder, pancreas and spleen Doppler ultrasound | 1 结肠癌肝转移 hepatic metastasis of colonic carcinoma | 2 肝可疑结节 Suspicious hepatic nodules | 3 胰腺可疑结节 Suspicious pancreatic nodules | 4 肾上腺可疑结节 Suspicious adrenal nodules | 5 肝硬化 liver cirrhosis | 6 腹膜后占位 Retroperitoneal mass | 7 肝原发癌 primary hepatocellular carcinomaHepatocellular carcinoma HCC | 8 腹腔占位 Celiac mass | 9 未见恶性征象或肝硬化 without malignancies or liver cirrhosis | 10 胆囊息肉 cholecystic polypus | |
| UR1 | 肾占位 | Renal lesion | 1 恶性征象  Suspected malignant | 2 无恶性征象  Without malignancies |  |  |  |  |  |  |  |  | |
| U5 | 腹部超声 | Liver, gallbladder, pancreas and spleen Doppler ultrasound | 1 肝占位 liver lesion/masses | 2 胰腺占位 pancreatic lesion/masses | 3 肾上腺占位 Adrenal mass | 4 腹腔腹膜后占位  Abdominal and retroperitoneal mass | 5 肾占位  Renal lesion/masses | 6 肝硬化 liver cirrhosis | 7 胆囊息肉 cholecystic polypus | 8 未见恶性 without malignancies | |  |  |
| US | 超声总计 | All Doppler ultrasound | 1 有可疑征象 Suspected malignant | 2 无可疑征象 Without malignancies | |  |  |  |  |  |  |  |  |
| Ctlung | CT胸部 | Lung CT | 1 肺癌可能（包括复发）suspected lung cancer (including recurrence) | 2 转移可能（包括骨转移）suspected metastasis (including bone metastasis) | 3 胸膜间皮瘤可能 suspected Pleural mesothelioma | 4 磨玻璃影 ground-glass opacity | 6 无以上征象 without previous lesions | |  |  |  |  |  |
| CT1 | 胸部CT | Lung CT | 1 肺癌可能 suspected lung cancer | 2 转移癌 suspected metastasis | 3 磨玻璃影 ground-glass opacity | 4 其它 others |  |  |  |  |  |  |  |
| CTL1 | 肺CT | Lung CT | 1 原发肺癌 primary lung cancer | 2 无原发肺癌征象 not primary lung cancer |  |  |  |  |  |  |  |  |  |
| CTPP | CT腹盆 | Abdominal and pelvic CT | 1 无异常 without abnormalities | 2 肝转移癌/肝占位 liver lesions/masses | 3 结肠癌 colon cancer | 4 肾癌 renal cancer | 5 卵巢恶性（囊腺癌）Ovarian malignancy (cystadenocarcinoma) | 6 腹腔腹膜后淋巴结转移 Abdominal and retroperitoneal lymph node metastasis | 7 动脉瘤 arterial aneurysm | 8 淋巴结追查 suspected enlarged lymph node | 9 肾或肾上腺占位追查 Renal or adrenal mass | 10 骨占位/破坏追查 Bone lessions/masses/destruction | 11 胰腺占位待查 pancreatic lesion/masses |
| CT2 | 腹盆CT | Abdominal and pelvic CT | 1 肝占位 liver lesions/masses | 2 结肠占位 colon lesions/masses | 3 肾/肾上腺占位 Renal or adrenal mass | 4 卵巢占位 ovarian mass | 5 胰腺占位 pancreatic lesion/masses | 6 骨占位 Bone lessions/mass | 7 其它/无恶性 other/not malignant | |  |  |  |
| Ctp2 | 腹盆CT | Abdominal and pelvic CT | 1 结直肠癌  Colorectal cancer | 2 无结直肠癌征象 not Colorectal cancer |  |  |  |  |  | |  |  |  |
| CTP3 | 腹盆CT | Abdominal and pelvic CT | 1 肾癌 renal cancer | 2 无肾癌征象 not renal cancer |  |  |  |  |  | |  |  |  |
| summary | 总结 | summary | 1 甲状腺乳头状癌 papillary thyroid carcinoma | 2 肺腺癌 lung adenocarcinoma | 3 乳腺癌 breast cancer | 4 肾透明细胞癌 clear cell carcinoma of kidney | 5 胃癌 gastric cancer | 6 结直肠癌 colorectal cancer | 7 卵巢癌 ovarian cancer | 8 结肠高级别上皮内瘤变 High-grade intraepithelial neoplasia of the colon | 9 白血病 leukemia | 10 肾癌合并肺癌 Renal cell carcinoma and lung cancer | 11 食管癌 esophageal cancer |
| outcome | 癌筛结果 | outcome | 1 确诊癌症 confirmed malignancy | 2 排除癌症 ruled malignancy | |  |  |  |  |  |  |  |  |
| ctype1 | 上消化道癌 | Cancer of the upper gastrointestinal tract | 1 确诊 confirmed | 2 排查 ruled |  |  |  |  |  |  |  |  |  |
| ctype2 | 结直肠癌 | Colorectal cancer | 1 确诊 confirmed | 2 排查 ruled |  |  |  |  |  |  |  |  |  |
| ctype3 | 乳腺癌 | Breast cancer | 1 确诊 confirmed | 2 排查 ruled |  |  |  |  |  |  |  |  |  |
| ctype4 | 甲状腺癌 | Thyroid cancer | 1 确诊 confirmed | 2 排查 ruled |  |  |  |  |  |  |  |  |  |
| ctype5 | 肺癌 | Lung cancer | 1 确诊 confirmed | 2 排查 ruled |  |  |  |  |  |  |  |  |  |
| Ctype6 | 肾癌 | Renal cancer | 1 确诊 confirmed | 2 排查 ruled |  |  |  |  |  |  |  |  |  |
| otc2 |  | outcome | 1 超声诊断的癌 cancer confirmed by ultrasound | 2 其它癌 others | 3 非癌 not malignant |  |  |  |  |  |  |  |  |
|  |  |  |  |  |  |  |  |  |  |  |  |  |  |
|  |  |  |  |  |  |  |  |  |  |  |  |  |  |
